# The Incremental Added Value of Including the Head in ^18^F-FDG PET/CT Imaging for Cancer Patients

**DOI:** 10.3389/fonc.2013.00071

**Published:** 2013-04-04

**Authors:** Amir G. Abdelmalik, Saud Alenezi, Razi Muzaffar, Medhat M. Osman

**Affiliations:** ^1^Division of Nuclear Medicine, Department of Radiology, Saint Louis UniversitySaint Louis, MO, USA; ^2^Department of Nuclear Medicine, Kuwait UniversityKhaldiya, Kuwait; ^3^Saint Louis VA Medical CenterSaint Louis, MO, USA

**Keywords:** ^18^F-FDG, PET/CT, brain imaging, PET/CT field of view, brain metastasis

## Abstract

**Purpose:** To assess the value of extending the routinely used base-of-skull (BOS) to upper-thigh field of view (FOV) to include the head on ^18^F-FDG PET/CT in cancer patients.

**Methods:** We retrospectively reviewed 1000 consecutive top-of-head to foot PET/CT studies. Abnormalities above BOS were categorized as unsuspected or known and were correlated with pathology, MRI/CT, and clinical follow-up.

**Results:** Of the 1000 patients, 102 (10.2%) had potentially significant findings above BOS. Of these, 70/102 (69%) were known and 32/102 (31%) were unsuspected. Of the patients with unsuspected findings, follow-up data was unavailable in 7/32 (22%) and abnormalities were confirmed in 25/32 (78%). Of the 25 confirmed unsuspected findings, 4/25 (16%) were false positives and 21/25 (84%) were true positives. Of these, 13/21 (62%) were confirmed metastatic, and 8/21 (38%) were benign. Unsuspected finding of brain metastasis changed the management in 11/13 (85%) and staging in 4/13 (31%).

**Conclusion:** Including the head in PET/CT FOV incidentally detected clinically significant findings in 2.1% (21/1000) of patients. The detection of previously unsuspected metastasis had significant impact on patient management and provided more accurate staging.

## Introduction

Positron emission tomography (PET) using fluorine-18-2-deoxy-d-glucose (^18^F-FDG) diagnoses, stages, and restages many cancers with an accuracy ranging from 80 to 90% (Czernin and Phelps, 2002).

In oncology, Whole Body (WB) PET/CT (positron emission tomography – computed tomography) is typically performed from the base-of-skull (BOS) to the pelvic-floor (Von Schulthess et al., 2006). The use of the term WB is misleading since the most commonly used field of view (FOV) does not include the brain/skull, and significant portions of upper and lower extremities. Even in melanoma patients, in whom top-of-head to the feet is the standard of care, routine inclusion of the head and extremities has been recently questioned (Niederkohr et al., 2007).

Brain metastasis is a common complication of cancer affecting 15–40% of patients (Grupta et al., 1999). These patients have a poor prognosis even in the absence of systemic disease, with a median survival time ranging from 9 to 18 months (Chidel et al., 1999). The most common primary cancers that metastasize to the brain in adults are lung (40%), breast, colon, renal cell carcinoma, and melanoma. In children, the most common are sarcoma and germ cell tumor. The cerebral cortex is the most common location for cerebral metastasis (80%) with multiple lesions in two-thirds of the patients (Vecht, 1998).

Our objective was to evaluate the incremental added value of extending the routinely used PET/CT FOV, BOS to upper-thigh, to include the head in imaging different cancer patients.

## Materials and Methods

### Patients

We retrospectively evaluated all available data for a total of 1000 consecutive patients referred for clinical evaluation of known or suspected malignancy and who had undergone top-of-head to bottom of feet PET/CT scans, the standard of care at our institution. The primary diagnosis was: lung (*n* = 314), head and neck (*n* = 228), gastrointestinal (*n* = 181), lymphoma (*n* = 113), breast (*n* = 39), and others (*n* = 125). Patients who were referred for melanoma staging or metastatic brain lesions of unknown primary were excluded from the study since the brain is typically included in these cases.

### PET/CT scanning

An intravenous 5.18 MBq/kg (0.14 mCi/kg) injection of ^18^F-FDG was administered after the patient had fasted at least 4 h. Patients sat in a quiet room without talking during the subsequent 60 min of the FDG uptake phase. Blood glucose level was <200 mg/dl in all patients. All scans were acquired using a PET/CT scanner (Gemini TF; Philips Medical Systems) with an axial co-scan range of 193 cm.

### CT scanning

The CT component of the PET/CT scanner consisted of a 64 slice multidetector helical CT with a gantry port of 70 cm. Parameters were as follows for 20–21 bed acquisitions: 120–140 kV and 33–100 mAs (based on body mass index), 0.5 s per CT rotation, pitch of 0.9 and 512 × 512 matrix. CT data were used for image fusion and the generation of the CT transmission map. In all patients, the arms were placed above the patient’s head for CT acquisition except in patients with head and neck cancers where the arms were placed at the patient’s sides. The CT images were obtained without oral or IV contrast.

### PET scanning and image processing

The PET component of the PET/CT scanner is composed of Lutetium-Yttrium Oxyorthosilicate (LYSO)-based crystals. Emission data were acquired on average for 20–21 bed positions (193 cm coverage, identical to the CT protocol). Emission scans were acquired at 1–2 min per bed position. The FOV was from the top-of-head to the bottom of feet in all patients. The three-dimentional (3D) WB acquisition parameters consisted of a 128 × 128 matrix and an 18 cm FOV with a 50% overlap. Processing consisted of the 3D Row Action Maximum Likelihood Algorithm (RAMLA) method (Browne and De Pierro, 1996). Total scan time per patient was 20–45 min.

### Image analysis

PET/CT images were retrospectively evaluated on Extended Brilliance workstation (Philips Medical Systems) by two board-certified Nuclear Medicine physicians. A log was kept to record cases of suspected lesions above the BOS. Final interpretation was reached by consensus. Data were categorized into two groups: known pathology and new, previously unidentified pathology above the BOS. Suspected pathology was correlated with surgical pathology, MRI and/or CT, or clinical follow-up. The impact on management and/or staging from the detection of unidentified pathology above the BOS was confirmed by a board-certified oncologist.

## Results

**Figure 1:**
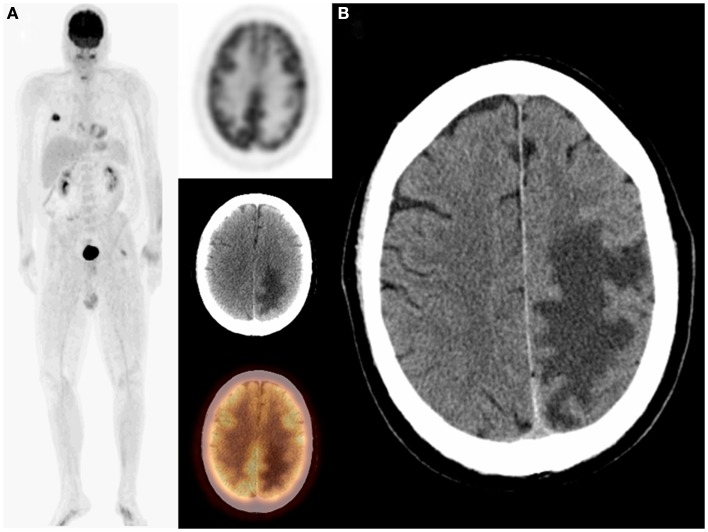
**Sixty-five-year-old female with a history of thyroid and metastatic breast cancers as well as schwannoma which was surgically resected**. On restaging PET/CT: MIP, transaxial PET, CT, and fused images **(A)** demonstrated an unsuspected finding of recurrent schwannoma in addition to metastatic breast cancer. PET/CT finding was confirmed on MRI **(B)**. The patient subsequently underwent surgical resection of the recurrent shwannoma.

Of the 1000 PET/CT cases, 102 (10.2%) patients had clinically significant PET/CT findings above the BOS. Of these, 70/102 (69%) patients were known or suspected to have pathology above the BOS based on clinical and radiographic data. Those were excluded from our final analysis. The remaining 32/102 patients (31%) had new unsuspected findings above the BOS. Follow-up data was not available for 7/32 (22%) patients. Abnormal findings were confirmed in the remaining 25/32 (78%) patients. Of these 25 patients, 4/25 (16%) were false positive and 21/25 (84%) were true positive. Metastasis was confirmed in 13/21 (62%; 12 male and 1 female, mean ages 62). Patient characteristics and the distribution of malignant lesions above the BOS are summarized in Table 1. The remaining 8/21 (38%) patients had confirmed benign pathology (Figure 1) above the BOS (four males and four females, mean ages 69). Patient characteristics are summarized in Table 2.

**Table 1:** **Patients with unsuspected finding of malignant lesions above the base-of-skull**.

No.	Primary	G	Age	Distribution of brain lesions	Stage with LWB	Stage with TWB	Change in management
1	Neuroendocrine of the cecum	M	44	Rt (right) frontoparietal	IV	IV	Unknown
2	Lung	M	67	Lt (left) temporal	IV	IV	whole brain (WB) radiation
3	Lung	M	65	Rt frontal	I	IV	surgical resection followed by WB radiation
4	Lung	F	80	Rt frontal and Lt temporoparietal	I	IV	Cyberknife therapy
5	Lung	M	65	Multiple	III	IV	WB radiation
6	Lung	M	65	Lt frontal	IV	IV	WB radiation
7	Lung	M	61	Rt frontal	IV	IV	Cyberknife therapy
8	Right nasal cavity	M	27	Rt parietal and Lt frontal	IV	IV	WB radiation
9	Lung	M	64	Rt frontal	IV	IV	WB radiation
10	Lung	M	63	Rt temporal	IV	IV	Cyberknife therapy
11	Mucoepidermoid tumor of right tongue	M	66	Lt frontal	IV	IV	Unknown
12	Lung	M	63	Rt frontal and parietal	IV	IV	WB radiation
13	Lung	M	70	Lt frontoparietal	I	IV	WB radiation

*LWB, limited whole body field of view from the base of the skull to the upper-thighs*.

*TWB, true whole body field of view from the top of the skull to the feet*.

**Table 2:** **Patients with unsuspected finding of benign lesions above the base-of-skull**.

Patient no.	Primary	Gender	Age	Benign finding	Change in management
1	Lung	M	63	Lipoma	Unknown
2	Lung	F	83	Infarction	No change
3	Lung	M	59	Pituitary macroadenoma	No change
4	Gastric	F	71	Pituitary macroadenoma	No change
5	Lung	M	79	Benign atrophy	No change
6	Thyroid/breast	F	65	Recurrent schwannoma	Resection of recurrent tumor
7	Abdominal liposarcoma	M	74	Infarction	Started on Aspirin
8	Squamous cell carcinoma of head and neck	F	61	Neurosarcoid	Unknown

**Figure 2:**
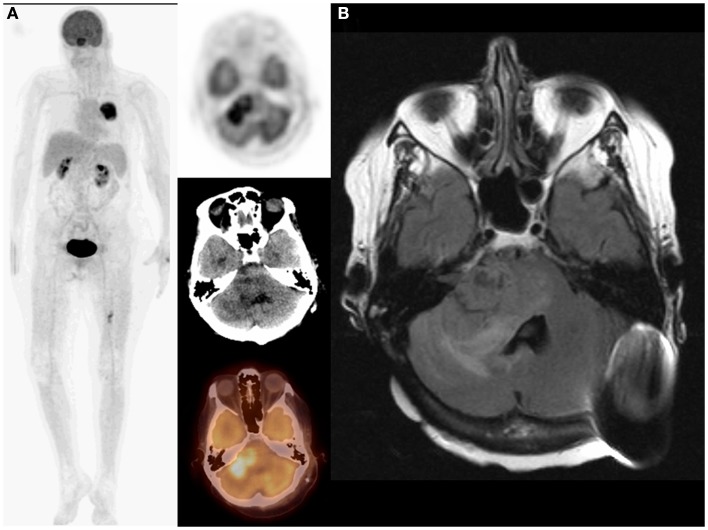
**Eighty-year-old female with non-small-cell lung cancer in the right lung status post Cyberknife therapy**. On restaging PET/CT: MIP (maximum intensity projection) and transaxial PET, CT, and fused images **(A)** demonstrated complete response to therapy in the right lung and a new left temporoparietal lobe lesion suspicious for metastasis. PET/CT finding was confirmed on MRI **(B)**. Adding the brain to the imaged field of view changed both staging (I–IV) and management as patient underwent whole brain radiation.

**Figure 3:**
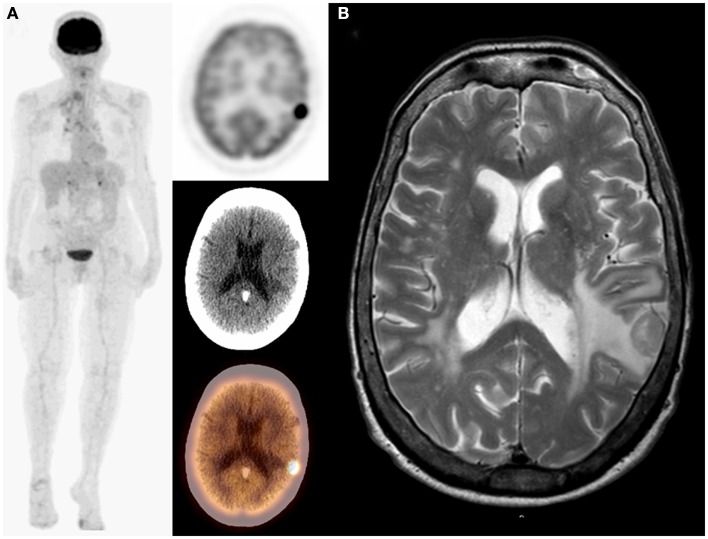
**Seventy-year-old male with bronchogenic carcinoma in the right lung**. On initial staging PET/CT: MIP, transaxial PET, CT, and fused images **(A)** demonstrated a single lesion in the right lung and unsuspected photopenia in the left frontoparietal lobe that corresponds to a hypodensity on CT suspicious for metastasis. PET/CT finding was confirmed on MRI **(B)**. Adding the brain to the imaged field of view changed both staging (I–IV) and management as patient underwent whole brain radiation.

Of significance, 2/13 patients (15%) with unsuspected metastases had their only malignant site in the brain; both with treated lung cancer (Figure 2). One patient had a left frontoparietal lobe lesion as the only metastatic site on initial staging PET/CT (Figure 3). Unsuspected finding of brain metastasis changed the management in 11/13 (85%) patients and upstaged 4/13 (31%) patients (Table 1).

**Figure 4:**
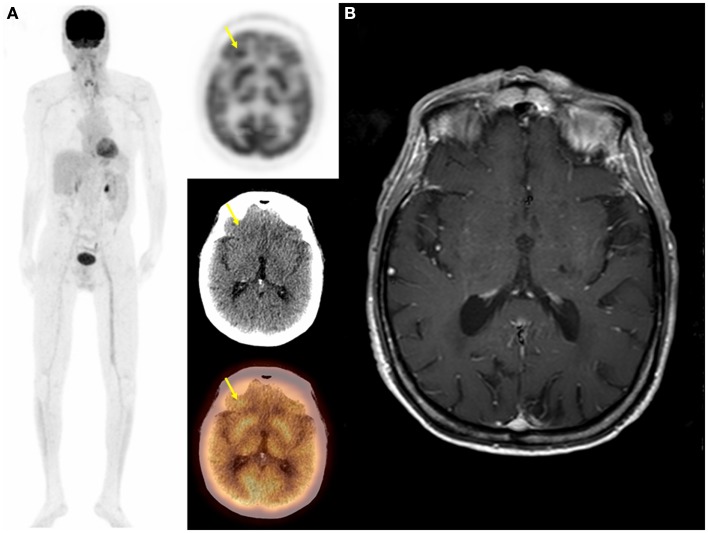
**Seventy-one-year-old male with metastatic squamous cell carcinoma of the head and neck**. On follow-up PET/CT: MIP, transaxial PET, CT, and fused images **(A)** demonstrated an unsuspected hypermetabolic focus is the right frontal lobe with no definite abnormality on CT in addition to metastatic head and neck cancer. Brain MRI was negative for metastasis **(B)**. In this case PET was falsely positive and the focal uptake in the right frontal lobe was thought to be due to artifact.

Positron emission tomography was false positive in 4/25 patients, two with focal sellar uptake, one with increased uptake in bilateral lentiform nuclei and thalamus, and one with focal right frontal lobe uptake (Figure 4). All of these patients had a normal follow-up brain MRI.

## Discussion

PET/CT technology has been in rapid evolution and dissemination. It is becoming a standard procedure in the management of many cancer patients. However, the FOV for WB-PET/CT imaging of cancer patients is not standardized and varies between institutions (Huston et al., 2010). The most commonly used WB-PET/CT FOV protocols only images from the BOS to the upper-thighs. A publication from the National Oncologic PET Registry (NOPR) showed that the FOV for PET was not recorded in 8.4% of PET/CT reports (Coleman et al., 2010). Furthermore, there has been significant variation among sites in individual descriptions of craniocaudal anatomic landmarks within this FOV. Beyer et al. (2011) reported that the term BOS was used to describe supraclavicular anatomic landmark in 53% of sites.

The routinely used FOV may underestimate the true extent of the disease by missing metastases to areas outside the typical BOS to upper-thigh FOV. Initial studies are beginning to appear in the literature documenting the added value of top-of-head to feet FDG PET imaging in some types of cancers (Nguyen et al., 2007). In a recent publication by NOPR evaluating the impact of dedicated brain PET on intended patient management, they concluded that dedicated brain PET was associated with similar net changes in intended management as in the overall NOPR cohort (Hillner et al., 2011). In a previous study, 4% of cancer patients who underwent top-of-head to feet PET/CT, had undetected malignant sites outside the typical BOS to upper-thigh FOV (Osman et al., 2010). The co-scan range for combined CT and PET imaging is approximately 145 cm for most of the different PET/CT scanner designs offered by the major vendors of medical imaging equipment (Townsend et al., 2004). Therefore, for most PET facilities, top-of-head to feet PET/CT imaging would require two separate image acquisitions with patient repositioning in between. However, including the head in the FOV would require only single image acquisition in all available PET/CT scanners.

In a similar previous study performed in 1026 patients, unidentified abnormal brain findings were detected in 1.1% (0.4% were malignant and 0.7% were benign) (Ludwig et al., 2002). In another older study performed with 273 patients, unidentified cerebral metastasis were only detected in 0.7% of patients (Larcos and Maisey, 1996). Both studies evaluated PET only images and therefore lacking the anatomic information provided by the CT portion of PET/CT. Furthermore, the impact of the unsuspected detection of brain lesions was not fully assessed in either study.

In our study, the presence of previously unidentified pathology above the BOS was confirmed in 2.1% of 1000 patients. Of those, malignant findings were confirmed in 1.3% (13/1000) and benign findings were confirmed in 0.8% (8/1000). A change in patient management was seen in 85% (11/13) of patients with new detection of malignancy above the BOS and upstaging in 31% (4/13). Also, the presence of previously unknown benign findings above the BOS resulted in change of patient management two of the eight patients (25%). We propose to extend the commonly used BOS to upper-thigh FOV to include the head as the standard of care in imaging cancer patients.

The sensitivity of PET is suboptimal in detecting brain metastases due to the intense physiologic background uptake in the brain and the hypometabolic nature of some brain metastases. Therefore, contrast-enhanced MRI has been reported to have higher sensitivity and specificity for brain metastases than PET (Rohren et al., 2003). Although guidelines from the National Comprehensive Cancer Network (NCCN) included brain MRI as part of the initial work-up of non-small-cell lung cancer, it has not yet been enforced (National Comprehensive Cancer Network, 2011). The decision to obtain a brain MRI is left to the oncologist and may only be recommended for symptomatic patients or patients with advanced disease. Furthermore, NCCN guidelines did not recommend brain MRI for restaging or surveillance of these patients unless they become symptomatic. In our study, 50% of patients with primary lung cancer and unsuspected brain metastasis had their unsuspected brain findings on restaging/surveillance PET/CT scans. In addition, two patients had brain lesions as the only abnormalities on PET/CT (Figure 1).

In our study, patients with lung cancer represented 67% of the patients with unsuspected pathology above the BOS. However, we cannot conclude with confidence that lung cancer is the only malignancy that would benefit most from including the head in the FOV. Such a conclusion would be fraught with potential pitfalls if the incidence and prevalence of various cancers are not taken into account. Also, selection bias represents an additional limitation since PET/CT scans are not routinely obtained in breast cancer patients at our institution.

Extending the BOS to upper-thigh FOV in PET/CT to include the head will only increase the scan time by 1–5 min. Furthermore, although the FDG dose would be the same, including the head will minimally increase the patient’s radiation dose in the low-dose non-contrast CT portion. Of importance, the Medicare reimbursement rates for top-of-head to foot and BOS to upper-thigh have equal technical fees ($1037.34) with a difference of $2.89 in professional fees ($127.74 vs. $124.8). Therefore, including the head in the imaged FOV in PET/CT would not represent any significant financial burden.

Our study is not without limitations. The prevalence of patient motion resulting from the increased scan time was not evaluated. In addition, the CT component of the PET/CT was done using lower-dose technique and without contrast. We are also unable to provide an estimate of the false negative rate of top-of-head to foot PET/CT FOV in this retrospective study. Furthermore, the impact of false positive results and related costs and anxiety was not addressed. Lastly, a dedicated brain PET-CT acquisition was not performed on these patients. However, our goal was not to assess specificity or sensitivity of extending the BOS to upper-thigh FOV to include the head, but rather document its potential added value.

## Conclusion

We propose that including the head in the PET/CT imaged FOV may offer additional benefit to cancer patients by detecting clinically significant findings. Detection of additional metastasis in these patients had significant impact on patient management and provided more accurate staging.

## Conflict of Interest Statement

The authors declare that the research was conducted in the absence of any commercial or financial relationships that could be construed as a potential conflict of interest.
